# Investigating the influence of adolescents' social and emotional skills on health behavior: a moderated mediation analysis

**DOI:** 10.3389/fpsyg.2025.1712176

**Published:** 2025-12-16

**Authors:** Cixian Lv, Zihan Wang, Haoran Cui, Kejun Zhang, Xiuli Wang, Xinghua Wang, Taghreed A. Alsudais, Shifei Duan

**Affiliations:** 1 School of Education Science, Qingdao University, Qingdao, Shandong, China; 2 Qingdao Shinan District Teaching and Research Center, Qingdao, Shandong, China; 3 College of Education and Human Development, Princess Nourah bint Abdulrahman University, Riyadh, Saudi Arabia; 4 College of Education, Zhejiang University, Hangzhou, Zhejiang, China

**Keywords:** social and emotional skills, health behavior, test and class anxiety, satisfaction with the interpersonal relationships, moderated mediation

## Abstract

**Background:**

In the context of heightened social competition and increasing academic pressure, promoting the healthy development of adolescents has become a critical concern. This study investigates the impact and mechanisms through which adolescents' social and emotional skills influence their health behavior. Specifically, we hypothesized that social and emotional skills would positively affect health behavior, with test and class anxiety mediating and satisfaction with interpersonal relationships moderating this relationship.

**Methods:**

Using data from the 2023 OECD Survey on Social and Emotional Skills, this study employed a moderated mediation model to analyze the relationships between variables. The sample consisted of 6,737 adolescents, comprising 3,454 10-year-olds (51.3%) and 3,283 15-year-olds (48.7%); 3,484 were boys (51.7%) and 3,253 were girls (48.3%). Key variables assessed included social and emotional skills (e.g., task performance, emotional regulation), health behaviors (e.g., diet, exercise, sleep), test and class anxiety, and satisfaction with interpersonal relationships. Statistical analyses included Harman's single-factor test for common method bias, correlation analysis, regression analysis, and PROCESS macro in SPSS to test mediation and moderation effects.

**Results:**

The study found that adolescents' social and emotional skills positively influence their health behavior, with a significant direct effect (β = 0.375, *p* < 0.001) and an indirect effect mediated by test and class anxiety [6.68% of total effect, 95% CI (0.017, 0.033)]. Satisfaction with interpersonal relationships moderated both the direct effect of social and emotional skills on health behavior and the reduction of test anxiety by these skills. Higher relationship satisfaction strengthened the direct effect (β = 0.340 vs. β = 0.250 for low vs. high satisfaction) and amplified the anxiety-reduction effect (β = −0.262 vs. β = −0.168 for high vs. low satisfaction).

**Conclusion:**

This study highlights the critical role of social and emotional skills in promoting healthy behaviors among adolescents and underscores the importance of interpersonal relationships in moderating these effects. The results provide insights for policymakers and educators to design interventions that foster both social and emotional skills and supportive relational environments to enhance adolescent wellbeing.

## 1 Introduction

With the increasing pressure of social competition and the academic burden, more and more problems such as anxiety and depression are occurring among adolescents (Fishstrom et al., 2022; Wiklund et al., 2012). These challenges not only hinder their learning and daily lives but can also have long-term consequences for their future development. Social and emotional skills are core non-cognitive abilities that adolescents acquire and apply in relation to self-adaptation and social development (Chernyshenko et al., 2018). Drawing on the Big Five personality model, the Organization for Economic Co-operation and Development (OECD) categorizes social and emotional skills into the following five dimensions: Task Performance, Emotional Regulation, Collaboration, Engaging with Others, and Open-Mindedness (OECD, 2021). Researches have shown that good social and emotional skills can promote adolescents to increase their academic engagement and improve their academic performance (Xiao et al., 2025), reduce bad behavior (Delvecchio et al., 2022), improve happiness, and encourage health performance (OECD, 2021). Therefore, social and emotional skills are regarded as important protective factors for the development of adolescent health. For example, research indicates that active coping strategies and adaptive cognitive emotion regulation were associated with academic achievement goals and academic performance (Li et al., 2024). Further strengthening this connection, a meta-analysis by Taylor et al. (2017) demonstrated that school-based Social and Emotional Learning (SEL) interventions significantly improved adolescents' healthy identity development and positive social behaviors, which are foundational to sustained health practices. Similarly, Bermejo-Martins et al. (2018), in a trial of a health education programme, found that enhancing social and emotional skills directly contributed to the adoption of healthier lifestyles in young children, underscoring the early and pivotal role of these skills.

However, there have been few studies in the past that have focused on how social and emotional skills affect health behavior and the role of students' satisfaction with the relationships in this process. Test and class anxiety is a prevalent issue among adolescents that can consume psychological resources and directly impede the consistent practice of health behaviors (Turk, 2025). Empirical evidence from large-scale international assessments, such as the Programme for International Student Assessment (PISA), confirms that academic anxiety significantly undermines adolescents' wellbeing and is negatively associated with health-promoting behaviors (OECD, 2017). It is thus a plausible mechanism through which social and emotional skills, which aid in stress management, might influence health outcomes. Besides, according to Bronfenbrenner's ecological systems theory (Bronfenbrenner, 1979), the effect of individual competencies is shaped by their immediate social context. Satisfaction with interpersonal relationships represents a key aspect of this microsystem. Research has shown that positive interpersonal relationships can cultivate a sense of belonging and self-worth, thereby creating a supportive environment that helps individuals cope with various challenges (Dost-Gözkan, 2022). It was therefore hypothesized that relationship satisfaction would moderate the pathways in our model, potentially amplifying the benefits of social and emotional skills by providing support and resources. Based on this, this article uses the data collected from 6,737 participants to explore the impact and mechanism of adolescent social and emotional skills on their health behavior, in order to provide policy recommendations for promoting healthy living among adolescents. Overall, this study is driven to address the following research questions:

(1) What impact do the social and emotional skills of adolescents have on their health behavior?

(2) Does test and class anxiety play a mediating role between social and emotional skills and health behavior?

(3) How does satisfaction with interpersonal relationships influence the connection between social and emotional skills and health behavior?

## 2 Literature review and research hypothesis

### 2.1 The definition of social and emotional skills

Social and emotional skills have gained significant attention in recent years due to their critical role in shaping mental health, academic success, and overall wellbeing in adolescents. The concept of social and emotional skills is rooted in the broader field of SEL, which emphasizes the development of self-awareness, self-management, social awareness, relationship skills and responsible decision-making (CASEL, 2003). Then combining the Big Five Personality Model, the OECD has identified several key dimensions of social and emotional skills, including Open-Mindedness, Emotional Regulation, Collaboration, Task Performance, and En-gaging with Others (OECD, 2021). The Big Five Personality Model provides a robust and widely recognized framework for conceptualizing and measuring fundamental dimensions of personality, which encompass openness to experience, conscientiousness, extraversion, agreeableness, and neuroticism. These traits represent basic tendencies that underlie and influence an individual's patterns of thought, emotion, and behavior, and the model has been extensively validated across different cultures and age groups (Laverdière et al., 2013; Soto et al., 2011). Building upon this model and prior literature, the OECD has adopted several key dimensions of social and emotional skills in its assessment framework, including Open-Mindedness (related to Openness), Emotional Regulation (related to low Neuroticism), Collaboration (related to Agreeableness), Task Performance (related to Conscientiousness), and Engaging with Others (related to Extraversion). The adoption of this framework is justified due to its strong empirical foundation, its capacity to comprehensively summarize individual differences in social and emotional skills, and its predictive validity for important life outcomes (Walton et al., 2023).

### 2.2 The impact of social and emotional skills on health behavior

Behavior is the external manifestation of a people based on their knowledge, and skills. Health behavior generally refers to various activities widely participated in daily life to promote physical and mental health, including refusing drinking and smoking, maintaining sufficient sleep, and engaging in regular exercise (Kim et al., 2024). A robust body of evidence (e.g., Chernyshenko et al., 2018; Karimzadeh et al., 2012) links stronger social and emotional skills to the adoption of these positive health behaviors, particularly during adolescence. Specifically, empirical studies have consistently shown that adolescents with higher levels of emotional regulation and self-control are less likely to engage in risky behaviors such as smoking, alcohol consumption, and bullying (Bermejo-Martins et al., 2018; Sancassiani et al., 2015). Moreover, adolescents who demonstrate stronger collaboration and social awareness tend to report higher engagement in prosocial and health-supportive behaviors, partly due to improved peer relationships and stress coping (Oberle et al., 2023). A study conducted on high school students (Maglica and Dorcic, 2015) showed that the Big Five personality, which corresponds to social and emotional skills, has a significant impact on high school students' alcohol and smoking behavior. In addition, a long-term longitudinal study has shown that non-cognitive abilities in adolescents have a significant impact on their middle-aged healthy quality of life. For instance, adolescent conscientiousness reduces the risk of cardiovascular disease in adulthood, while agreeableness enhances health-related quality of life and decreases physiological stress (Atkins et al., 2020). Given this, this article proposes the following hypothesis.

H1: Adolescents' social and emotional skills have a positive effect on their health behavior.

### 2.3 The mediating role of test and class anxiety

Test and class anxiety is a negative emotional state experienced by students during academic activities (Collie et al., 2017), linked to fears or worries about potential adverse outcomes or failure during assessments (Zeidner, 2007). It commonly emerges when students feel that the challenges of the testing environment surpass their capabilities, and it correlates inversely with academic achievement, encompassing both standardized assessments and college entrance examinations (Von der Embse et al., 2018). Test and class anxiety is a multifaceted issue influenced by individual, environmental, and academic factors. Individual factors include personality traits like neuroticism and conscientiousness, as well as cognitive aspects such as self-efficacy and stress management skills (Pekrun et al., 2009; Rahafar et al., 2016). Environmental factors, such as parental expectations, teacher support, and peer relationships, also play a significant role in shaping students' anxiety levels (Bermejo-Martins et al., 2019; OECD, 2017). Academically, high-stakes testing, perceived difficulty of exams, and the value placed on academic success are key contributors to test anxiety (Garnefski and Kraaij, 2018; Olatunji et al., 2023).

Based on previous research, social and emotional skills can affect students' physical and mental health (Bermejo-Martins et al., 2019), and this impact may be mediated by test and class anxiety, as emotions are closely related to health performance. Specifically, the improvement of social and emotional skills may first alleviate students' test and class anxiety levels. For example, Wang and Qian (2024) found that adolescents with higher levels of social and emotional skills reported lower levels of test anxiety in a large-scale SSES dataset, indicating that stronger socio-emotional competencies may contribute to reduced academic anxiety. The value theory of academic emotional control holds that academic emotions stem from an individual's sense of control and value toward their studies (Pekrun et al., 2010). And this sense of academic control is highly correlated with task performance of social and emotional skills. Therefore, if an individual's perceived academic value far exceeds their own task performance, the individual will experience test and class anxiety (Rahafar et al., 2016; Nguyen et al., 2013). Secondly, the alleviation of test and class anxiety will promote students' health behavior. Anxiety's interpretation plays a significant role in how individuals perceive and evaluate challenging events and situations (Strack and Esteves, 2015). From the perspective of the impact of test and class anxiety, long-term exposure to severe academic anxiety not only affects an individual's academic life, but may also trigger emotional and sleep disorders, and even lead to suicidal behavior (Pekrun et al., 2009). The 2015 Programme for International Student Assessment (PISA) survey on students' quality of life, which specifically assessed 15-year-olds, found that academic anxiety undermines adolescents' happiness, triggers negative emotions, and harms health behaviors (OECD, 2017). In view of this, this article proposes the following hypothesis.

H2: Test and class anxiety plays a mediating role in the relationship between social and emotional skills and health behavior.

### 2.4 The moderating effect of satisfaction with the relationships

Human attitudes, skills, and behaviors are often shaped by both internal personal factors and external environmental factors (Schunk and DiBenedetto, 2020). Satisfaction with interpersonal relationships is viewed as a student's subjective evaluation of the quality of their key social connections, specifically with parents or guardians, friends, classmates, and teachers. Relationship satisfaction is crucial not only for adolescents' social and emotional skills but also for how these skills translate into adaptive outcomes.

Firstly, social and emotional skills are foundational for establishing and maintaining supportive relationships (Durlak et al., 2011). Adolescents with strong social and emotional skills are better equipped to resolve conflicts, express emotions constructively, and elicit positive social feedback, thereby enhancing satisfaction with their interpersonal relationships (Yu and Hu, 2024; Zhang et al., 2022). Conversely, satisfying and supportive relationships reinforce emotional stability and provide a safe context for practicing and refining social and emotional skills [Collaborative for Academic, Social, and Emotional Learning (CASEL), 2023]. Thus, social and emotional skills and relationship satisfaction function in a reciprocal and mutually reinforcing manner. Secondly, the stress-buffering perspective further suggests that high-quality relationships can moderate the impact of stressors and individual traits on psychological and behavioral outcomes (Cohen and Wills, 1985). Adolescents with greater satisfaction in their relationships are likely to experience enhanced emotional security and a sense of belonging, which can protect against anxiety, loneliness, and maladaptive behaviors (Alessandri et al., 2009; Caprara et al., 2006; Liu et al., 2023). Empirical evidence supports this. For example, Zhang et al. (2022) showed that perceived peer and parental support reduced the negative effects of stress on adolescents' emotional and behavioral adjustment, while Yu and Hu (2024) found that emotional competence and social engagement were associated with higher resilience when social relationships were supportive. On April 19, 2017, the OECD's PISA 2015 Results (Volume III): Students' Wellbeing (OECD, 2017) reported that student anxiety over homework and exams stems more from perceived teacher and school support than from school hours or test frequency. Campus conflicts were also a key source of stress. Based on the theoretical and empirical foundations outlined above, a reciprocal and reinforcing relationship exists between social and emotional skills and relationship satisfaction. Furthermore, high-quality relationships are posited to serve as a critical external resource that moderates the link between internal competencies and outcomes through a stress-buffering mechanism. Given these findings, this study proposes hypotheses H3 and H4.

H3: Satisfaction with the relationships positively moderates the direct effect of social and emotional skills on health behavior.

H4: Satisfaction with the relationships positively moderates the indirect effect of social and emotional skills on health behavior through testing and class anxiety.

**Figure 1:**
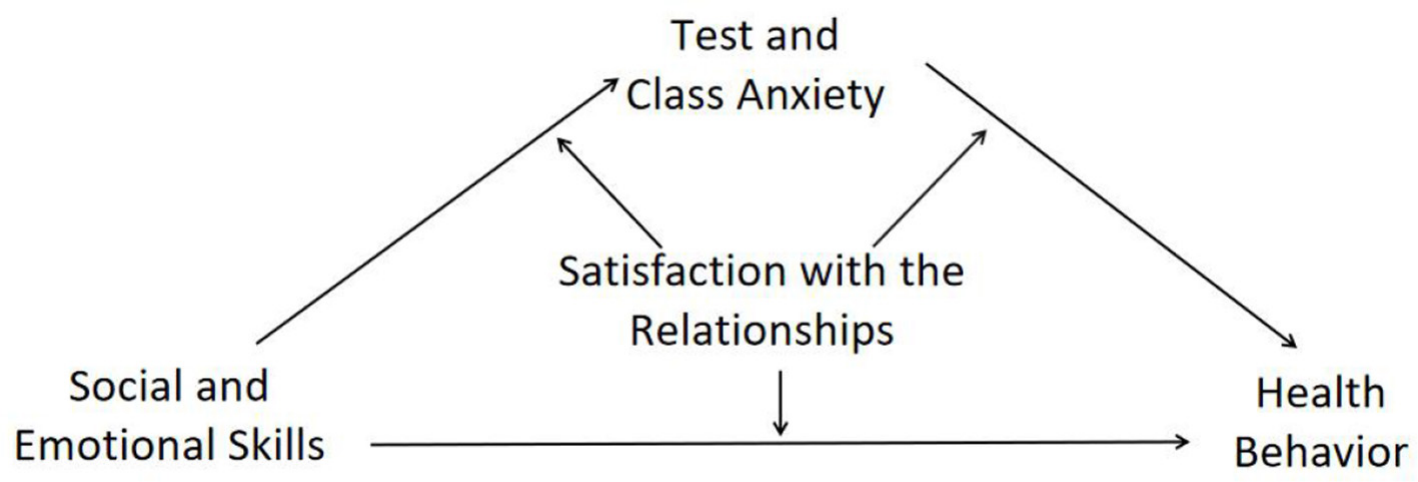
Theoretical model of a moderated mediation.

In summary, this study initially constructs a moderated mediation model (see Figure 1) to explore the impact and mechanism of adolescent social and emotional skills on health behavior.

## 3 Research methodology

### 3.1 Data source and participants

This study utilized the data from the OECD's 2023 Survey on Social and Emotional Skills (SSES), conducted at a city in China. The SSES is a comprehensive international survey designed to assess the development and influencing factors of social and emotional skills among students at two key developmental stages: 10-year-olds (representing late childhood) and 15-year-olds (representing early adolescence). These stages are characterized by significant physiological, psychological, and social transitions, during which socio-emotional development is particularly susceptible to academic pressure and peer influence. While the SSES collects a wide array of data from multiple sources, including students, parents, teachers, and school principals, this study focuses exclusively on a subset of student-reported variables, selected in accordance with our research hypotheses and theoretical framework. Data collection was administered by the OECD following a rigorous, standardized international protocol to ensure cross-national comparability. This process was conducted electronically, with students completing all self-report measures on computers during dedicated school sessions. A stratified two-stage cluster sampling design was employed to obtain a representative student sample. In the first stage, 77 primary schools and 75 secondary schools were selected based on stratification variables such as funding source (public/private), socioeconomic level of the school location, and school type. In the second stage, up to 50 eligible students were randomly selected from each participating school; if fewer than 50 were available, all eligible students were included. After excluding cases with missing values on key variables, the final analytical sample comprised 6,737 adolescents. As summarized in Table 1, the sample included 3,454 students aged 10 (51.3%) and 3,283 aged 15 (48.7%), with 3,484 males (51.7%) and 3,253 females (48.3%).

**Table 1:** Sample distribution.

**Cohort**	**Female**	**Male**	**Total**
10-years-old	1,686	1,768	3,454
15-years-old	1,567	1,716	3,283
Total	3,253	3,484	6,737

### 3.2 Variables

#### 3.2.1 Social and emotional skills

The SSES 2023 assessment framework for social and emotional skills is based on the Big Five Model and includes five key dimensions: Open-Mindedness, Emotional Regulation, Collaboration, Task Performance, and Engaging with Others. Each dimension is further divided into three to four sub-skills, with each sub-skill assessed through nine questions rated on a five-point scale ranging from 0 (“Strongly Disagree”) to 4 (“Strongly Agree”). To ensure data comparability, this study employs the Generalized Partial Credit Model (GPCM) from Item Response Theory (IRT) for parameter estimation. Initial capability values are calculated using the Weighted Likelihood Estimate (WLE) algorithm and are then transformed into standard scores with a mean of 500 and a standard deviation of 100 through linear transformation based on all SSES samples. The final scores of various social and emotional skills calculated by the above methods are shown in Table 2. The reliability of the measures is supported by high Cronbach's alpha coefficients for the five dimensions: Task Performance (0.922), Emotional Regulation (0.940), Collaboration (0.910), Engaging with others (0.911), and Open-Mindedness (0.888).

**Table 2:** Final score for sub-skills of social and emotional skills.

**Sub-skills**	**Min**	**Max**	**M**	**SD**
Assertiveness	−101.283	1145.234	560.999	551.216
Creativity	29.945	1014.976	579.091	555.228
Curiosity	32.898	1067.382	608.652	586.477
Emotional control	65.818	970.906	567.078	555.465
Empathy	49.137	1050.584	628.794	607.856
Energy	−121.718	1063.198	553.231	542.117
Optimism	186.658	878.156	576.998	568.065
Persistence	105.920	978.510	623.319	606.028
Responsibility	239.109	1020.291	634.348	616.839
Self-control	−93.481	1196.068	604.432	579.928
Sociability	75.415	991.679	567.416	555.429
Stress resistance	72.096	967.791	538.294	526.087
Tolerance	−43.569	1207.892	590.347	568.803
Trust	−62.733	1116.284	599.622873	588.694
Motivation	222.776	1048.936	609.379984	591.891

We chose social and emotional skills as a holistic independent variable rather than individual sub-skills because these skills are inherently interconnected and mutually reinforcing, as demonstrated by their integration during adolescence. The correlation coefficients between sub skills are shown in Table 3. The OECD framework further supports this holistic view, emphasizing that the collective influence of these skills on wellbeing and development was greater than the sum of their parts (OECD, 2021). Thus, examining social and emotional skills holistically provided a more comprehensive and impactful understanding of their influence on adolescent health behavior. To generate the total score of social and emotional skills, we add the scores of five dimensions of social and emotional skills and divide by 100.

**Table 3:** The correlation between the five dimensions of social and emotional skills.

**Dimension**	**1**	**2**	**3**	**4**
1. Task performance				
2. Emotional regulation	0.770^***^			
3. Collaboration	0.623^***^	0.616^***^		
4. Open-mindedness	0.739^***^	0.683^***^	0.639^***^	
5. Engaging with others	0.717^***^	0.737^***^	0.596^***^	0.709^***^

^***^p < 0.001.

#### 3.2.2 Health behavior

Health Behavior consists of 5 items: (1) eating breakfast, (2) eating vegetables and fruits, (3) exercising for no less than 20 min, (4) sleeping for more than 8 h, (5) smoking or drinking alcohol (only for 15-year-old students, reverse scoring), students answer from “1 = Never” to “5 = Every day”. This scale was developed drawing on the international survey Health Behavior in School-aged Children (HBSC) study (Inchley et al., 2018). The higher the score, the healthier the student's lifestyle. The internal consistency of this scale in the current sample was acceptable (α = 0.75). Confirmatory Factor Analysis (CFA) supported the unidimensional structure of the scale, with good model fit indices (CFI = 0.930, SRMR = 0.032), providing evidence for its construct validity.

#### 3.2.3 Test and class anxiety

The Test and Class Anxiety scale was adapted from the conceptual framework of the Test Anxiety Inventory, it consists of five items, covering two parts: learning anxiety and exam anxiety (Benson and El-Zahhar, 1994). Students are asked to rate their level of agreement with questions such as “I often worry that exams will be difficult for me” and “Even if I am well-prepared for exams, I feel very anxious.” The answer options are scored from “1 = Strongly disagree” to “5 = Strongly agree,” with higher scores indicating higher levels of test and class anxiety. The scale showed good internal consistency in our sample (α = 0.93). CFA results indicated excellent model fit (CFI = 0.932, SRMR = 0.045), confirming its strong construct validity.

#### 3.2.4 Satisfaction with the relationships

Satisfaction with the Relationships was measured using a scale adapted from the multidimensional life satisfaction framework (Huebner and Gilman, 2002). The scale consists of four items assessing students' satisfaction with their (1) parents or guardians, (2) friends, (3) classmates, and (4) teachers. Students answer from “0 = completely dissatisfied” to “10 = completely satisfied,” and finally divide their answers into four categories based on their scores: “dissatisfied” (0–4 points), “basically satisfied” (5–6 points), “satisfied” (7–8 points), and “very satisfied” (9–10 points). The higher the score, the higher the student's interpersonal satisfaction with the relationships. The scale showed adequate psychometric properties in the current sample. The internal consistency was acceptable (α = 0.85). Regarding construct validity, the CFI (0.969) and SRMR (0.031) provide strong evidence for the hypothesized factor structure.

## 4 Empirical results analysis

### 4.1 Common method bias test

Since all items in this study were self-reported by students, there is a potential risk of common method bias. To address this, Harman's single-factor test was employed using exploratory factor analysis with all measurement items included. The results indicated that the first unrotated factor explained 32.588% of the total variance, which is below the recommended threshold of 40% (Podsakoff et al., 2003). This suggests that common method bias is unlikely to be a serious concern in the present data, and further analysis is deemed appropriate.

### 4.2 Descriptive statistics and correlation analysis

Table 4 presents the mean, standard deviation, and correlation coefficient matrix of the main variables in this study *The data reveal several significant relationships. First, social and emotional skills show a significant correlation with test and class anxiety, health behavior, and interpersonal satisfaction. Specifically, there is a significant negative correlation between social and emotional skills and the mediator variable, test and class anxiety, indicating that individuals with higher social and emotional skills tend to experience lower levels of test and class anxiety. Second, a significant negative correlation is observed between test and class anxiety and health behavior, suggesting that higher levels of test and class anxiety are associated with poorer health behavior. Lastly, interpersonal satisfaction, serving as a moderating variable, is significantly correlated with the other key variables. Overall, these correlations highlight the potential for further testing of mediation and moderation effects*.

**Table 4:** Descriptive statistics and correlation analysis of major variables.

**Variables**	**M**	**SD**	**Min**	**Max**	**1**	**2**	**3**
1. Social and emotional skills	88.420	13.241	25.81	156.87			
2. Test and class anxiety	46.318	11.349	22.333	71.677	−0.363^***^		
3. Health behavior	56.442	11.666	−4.7149	76.613	0.462^***^	−0.271^***^	
4. Satisfaction with the relationships	56.455	13.430	7.069	80.614	0.564^***^	−0.318^***^	0.428^***^

^***^p < 0.001.

### 4.3 The impact of social and emotional skills on health behavior: the mediating role of test and class anxiety

**Figure 2:**
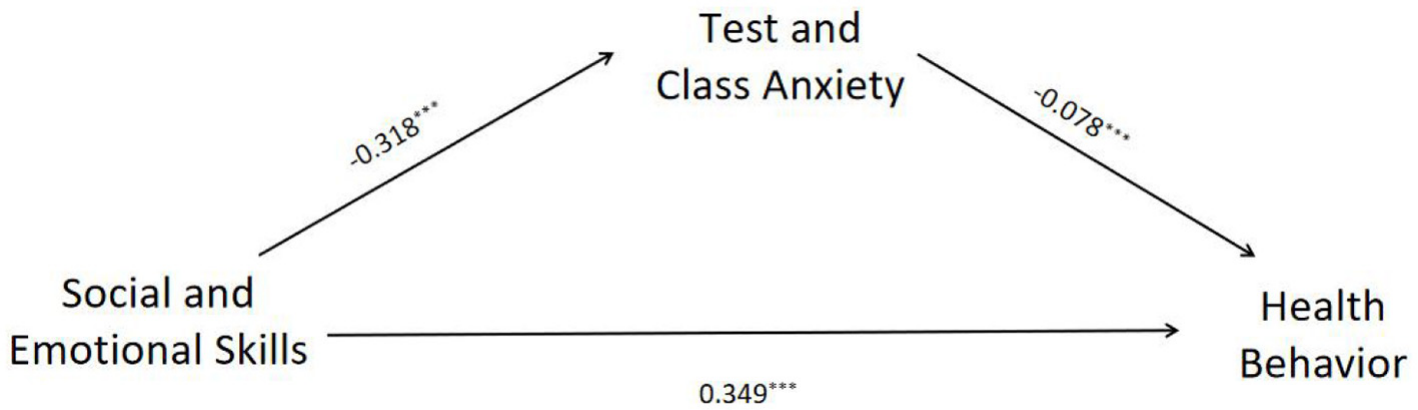
Regression analysis of the mediating effect. ***p < 0.001.

Firstly, we examined the direct impact of adolescents' social and emotional skills on their health behavior. The full model, which included covariates (gender, socio-economic status, and cohort), explained 26.6% of the variance in health behavior (*R*^2^ = 0.266), with a large effect size (Cohen's *f*^2^ = 0.36). This demonstrates the robust and independent contribution of social and emotional skills to health outcomes, even after accounting for demographic and contextual factors. Adolescents' social and emotional skills demonstrate a significant positive impact on their health behavior, as indicated by standardized regression coefficients (β = 0.375, *p* < 0.001), suggesting that a one-standard-deviation increase in social and emotional skills corresponded to a 0.375 standard-deviation improvement in health behavior. Following Cohen's (1992) conventions for effect sizes, this represents a medium-to-large effect, indicating a substantial and practically meaningful relationship. To further explore this relationship, Model 4 of the PROCESS macro in SPSS was used to test the mediating effect of test and class anxiety, while controlling for gender, age, and socioeconomic status. A bootstrap sample size of 2,000 was selected, with a 95% confidence interval. The path coefficients for test and class anxiety as mediators between social and emotional skills and health behavior are presented in Figure 2.

According to Table 5, the bootstrap 95% confidence interval for the impact of social and emotional skills on health behavior and the mediating effect of test and class anxiety does not include 0, indicating that social and emotional skills not only have a direct effect on health behavior, but also have a mediating effect on health behavior through the variable of test and class anxiety. The mediation analysis revealed that test and class anxiety accounted for 6.68% of the total effect [indirect effect: 0.025, 95% CI (0.017, 0.033)]. This completely standardized indirect effect is considered small in magnitude (Preacher and Kelley, 2011), yet it confirms a statistically significant and theoretically important pathway through which social and emotional skills influence health behavior. The results of these data support H1 and H2.

**Table 5:** Regression analysis of the mediating effect.

**Effect**	**Effect value**	**SE**	**LLCI**	**ULCI**	**Effect size**
**Social and emotional skills**→**test and class anxiety**→**health behavior**					
Total effect	0.374	0.011	0.352	0.396	
Direct effect	0.349	0.012	0.326	0.373	93.32%
Indirect effect	0.025	0.004	0.017	0.033	6.68%

LLCI, Lower Limit of Confidence Interval; ULCI, Upper Limit of Confidence Interval; Covariates: gender, cohort, socio-economic status.

### 4.4 The impact of social and emotional skills on health behavior: a moderated mediation test

We employ a hierarchical testing process for analyzing a mediation model with moderation. In the first step (Model 1), the relationship between social and emotional skills and students' health behavior is tested to determine whether this direct effect is significant. This step also establishes a simple moderation model to examine whether satisfaction with relationships moderates the direct effect. The second step (Model 2) evaluates whether social and emotional skills significantly influence students' test and class anxiety, while considering whether this effect is moderated by satisfaction with relationships. Finally, the third step (Model 3) investigates how social and emotional skills and test/class anxiety influence students' health behavior and whether satisfaction with relationships moderates these pathways. The parameter results for each model are summarized in Table 6.

**Table 6:** The relationship between social and emotional skills and health behavior: moderated mediation effect.

**Independent variable**	**Model 1: health behavior**	**Model 2: test and class anxiety**	**Model 3: health behavior**
	β **(95%** ***CI*****)**	β **(95%** ***CI*****)**	β **(95%** ***CI*****)**
Social and emotional skills (X)	0.295^***^ [0.267, 0.323]	−0.215^***^ [−0.245, −0.184]	0.281^***^ [0.253, 0.309]
Satisfaction with the relationships (U)	0.205^***^ [0.180, 0.230]	−0.142^***^ [−0.170, −0.115]	0.197^***^ [0.172, 0.222]
X^*^U	−0.045^***^ [−0.064, −0.027]	−0.047^***^ [−0.067, −0.027]	−0.052^***^ [−0.072, −0.032]
Test and class anxiety (W)			−0.058^***^ [−0.081, −0.036]
W^*^U			−0.012 [−0.032, 0.008]
*R* ^2^	0.297	0.179	0.302
*F*	464.088^***^	236.938^***^	352.142^***^

^***^p < 0.001.

**Figure 3:**
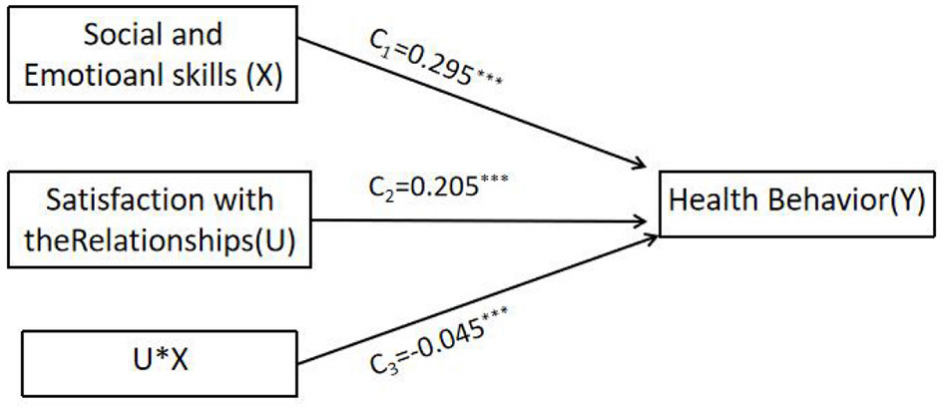
Model 1 tests whether the direct effect is moderated. ***p < 0.001.

**Figure 4:**
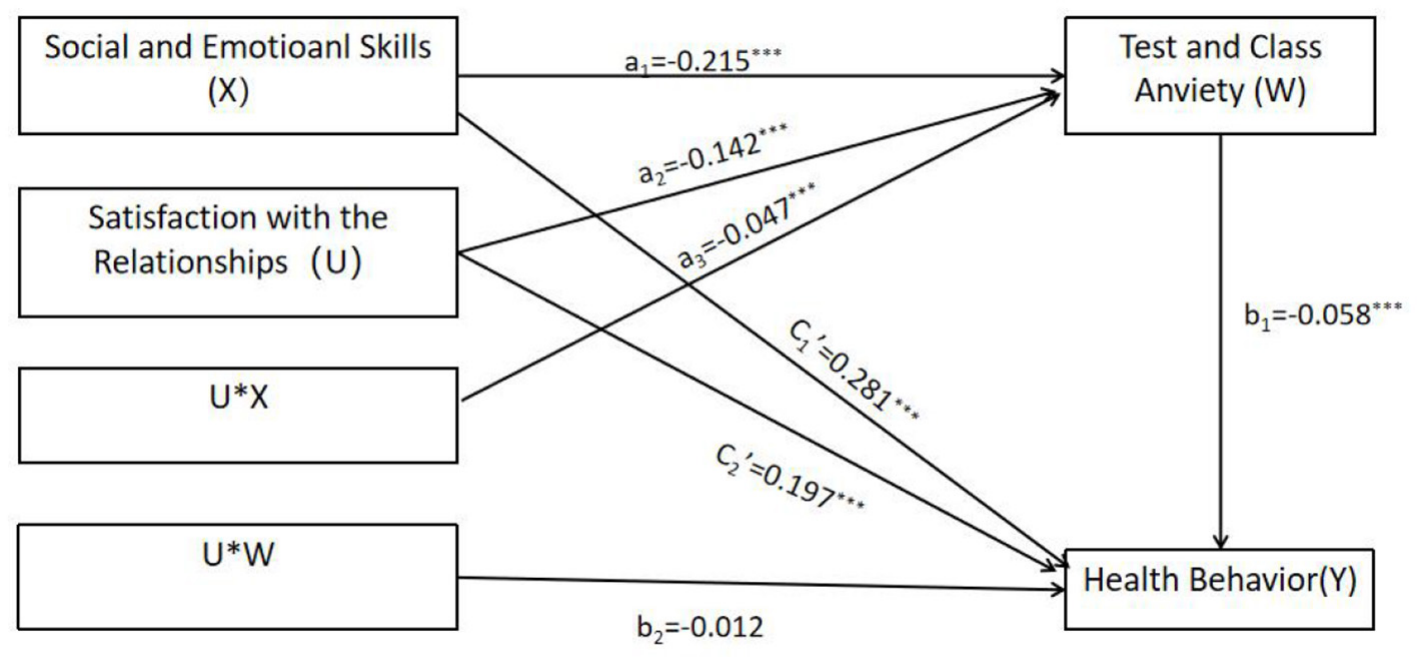
Model 2 and model 3 test whether the mediating effect is moderated. ***p < 0.001.

As shown in Figure 3, in Model 1, social and emotional skills have a significant positive impact on students' health behavior (*c*_1_ = *0.295, t* = *20.791, p*<*0.001*), and the direct effect of social and emotional skills on health behavior is moderated by satisfaction with the relationships. As shown in Figure 4, in Model 2, social and emotional skills have a significant negative impact on test and class anxiety (*a*_1_ = −*0.215, t* = −*13.900, p*<*0.001*), and satisfaction with the relationships has a significant moderating effect on the pathway. In Model 3, student test and class anxiety has a significant negative impact on health behavior (*b*_1_ = −*0.058, t* = −*5.117, p*<*0.001*). However, the predictive effect of the interaction term between satisfaction with the relationships and test and class anxiety on health behavior is not significant, indicating that the moderating effect of satisfaction with the relationships on the path between test and class anxiety and health behavior is not valid. Combining Model 2 and Model 3, it can be seen that adolescent social and emotional skills have a negative impact on test and class anxiety, while in Model 3, test and class anxiety have a negative impact on health behavior. Moreover, in Model 3, the impact of social and emotional skills on health behavior is still significant, indicating that test and class anxiety partially mediates the relationship between social and emotional skills and health behavior. The mediating effect of social and emotional skills (X) on health behavior (Y) through test and class anxiety (W) is *b*_1_(*a*_1_+*a*_3_U) = 0.012 + 0.003U, with a moderated mediation index of 0.003. When *U* values are 1, 0, and −1, the mediating effects are 0.015, 0.012, and 0.009, respectively. In summary, the direct impact of social and emotional skills on health behavior, as well as their mediating effect through testing and class anxiety, is influenced by satisfaction with relationships during the initial phase of the pathway.

**Figure 5:**
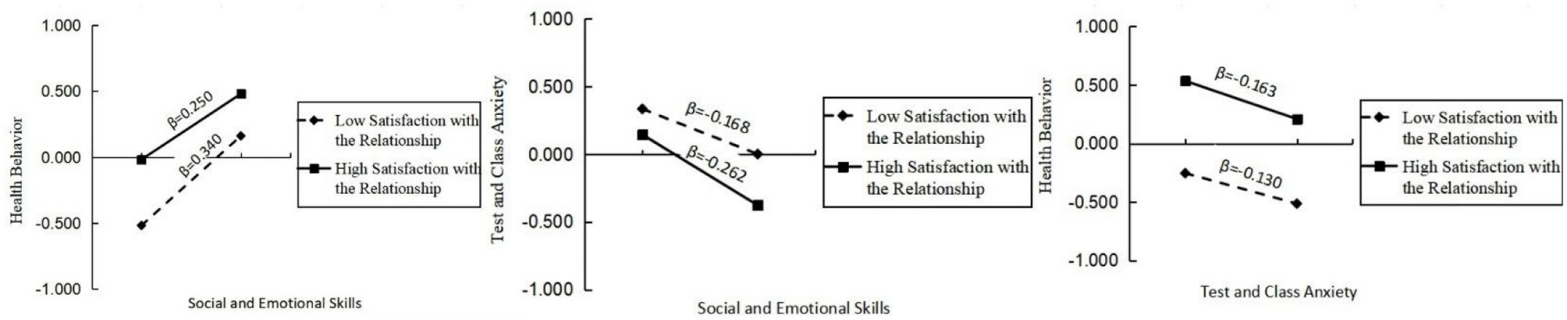
Simple slope test.

To better illustrate the moderating effect, a simple slope test was conducted, and the results are summarized below (see Figure 5). The findings indicated that social and emotional skills significantly influenced both health behaviors and test/class anxiety in both groups. Satisfaction with interpersonal relationships significantly moderated the direct effect of social and emotional skills on health behavior. For adolescents with low relationship satisfaction (M-1 SD), the direct effect was stronger (β = 0.340, *p* < 0.001). For those with high relationship satisfaction (M+1 SD), the effect diminished (β = 0.250, *p* < 0.001), possibly due to competing time investments in social interactions over health routines. Satisfaction with relationships amplified the first stage of the mediation pathway (social and emotional skills → test anxiety). Among adolescents with high satisfaction, social and emotional skills reduced anxiety more effectively (β = −0.262, *p* < 0.001) compared to those with low satisfaction (β = −0.168, *p* < 0.001). This suggests that supportive environments magnify the protective role of social and emotional skills against anxiety. However, the moderating effect on the second stage (anxiety → health behavior) was non-significant, indicating that relationship satisfaction primarily buffers anxiety formation rather than its behavioral consequences.

## 5 Discussion

This study develops a moderated mediation model to examine how adolescents' social and emotional skills influence their health behavior through the mediating role of test and class anxiety. Additionally, it explores how relationships with others moderate these pathways from a relational perspective. The findings highlight that social and emotional skills, as a key non-cognitive ability, play a vital role in fostering healthy behaviors among adolescents. Those with stronger social and emotional skills are better equipped to manage test and class anxiety and handle various pressures, leading to healthier habits such as regular physical activity, balanced dietary choices, and effective time management. Moreover, positive interpersonal relationships provide essential social support, help adolescents build a positive self-concept, and enhance their sense of social belonging, thereby further strengthening their social and emotional skills. This study offers theoretical guidance for fostering harmonious interpersonal relationships, alleviating academic anxiety, and promoting healthier behavior among students.

### 5.1 Social and emotional skills has a significant positive impact on adolescents' health behavior

Research find that social and emotional skills have a significant direct predictive effect on adolescents‘ health behavior, confirming research Hypothesis 1. On the one hand, individuals with higher social and emotional skills are more likely to maintain positive self-care activity (Martins et al., 2010). For example, when students have higher task performance, they tend to be more self-disciplined and have perseverance, and form healthy lifestyle habits (Strickhouser et al., 2017), such as regular exercise, healthy diet, and adequate sleep. These are key factors in maintaining physical health and preventing diseases. In addition, a randomized controlled trial of a school-based social-emotional and character-development programme (targeting adolescents) demonstrated modest but meaningful improvements in health-behavior indices (personal hygiene, healthy eating and exercise) in a youth sample in low-income communities (Bavarian et al., 2016). On the other hand, the development of social and emotional skills not only promotes the formation of healthy habits, but also enhances individuals' awareness and intrinsic motivation of the importance of health. Individuals with high levels of social and emotional skills are able to understand the behavior and reactions of others, respond appropriately, and exhibit behaviors that are beneficial to themselves (Bogg and Roberts, 2004). For example, in a study of 743 secondary school students, higher emotional intelligence and prosocial behavior were directly associated with healthier lifestyle dimensions (including diet, meal-timing) and lower involvement in substance use (González Moreno and Molero Jurado, 2024). In the domain of substance use refusal specifically, adolescent-focused research has shown that greater empathy and self-regulatory skills in early adolescents (6th−8th grade) were negatively associated with past-30-day drug use via the mediating role of refusal self-efficacy (Nguyen et al., 2011). Taken together, these adolescent-sample studies support our finding that stronger social and emotional competencies among adolescents are linked with more favorable health-behavior profiles, including more regular physical activity, healthier diet and sleep, and less substance use.

### 5.2 The mediating role of test and class anxiety between social and emotional skills and health behavior

Test and class anxiety, a common emotional experience among students, significantly affects their physical and mental health, as well as academic performance. This study indicates that test and class anxiety partially mediates the relationship between social and emotional skills and health behavior, thus supporting Hypothesis 2. In other words, social and emotional skills can improve adolescents' health by alleviating their test and class anxiety. Firstly, improving social and emotional skills can effectively alleviate testing and class anxiety in adolescents. Empirical evidence from middle school students confirms that students with strong social and emotional skills are better at managing their own emotions and planning their exam preparation systematically (Wang and Qian, 2024). This enables them to cope with the pressures and challenges of the learning process in a healthier way, with a more positive mindset, ultimately reducing the negative impact of stress (Beattie et al., 2010). Recent findings by Huo and Ning (2024) further illustrate the mechanism underlying this effect: emotional control and stress resistance function as central nodes within the socio-emotional skills network, meaning that improvements in these core competencies can generate system-wide benefits. This network-based perspective reinforces our mediating results by suggesting that strengthening key regulatory skills simultaneously enhances multiple interrelated socio-emotional processes, thereby enabling adolescents to regulate stress responses more efficiently and experience lower levels of test and class anxiety. Secondly, the alleviation of test and class anxiety can promote students' health behavior performance. Anxious adolescents tend to use more inadaptable coping strategies to cope with negative life events (Garnefski and Kraaij, 2018), such as smoking, drinking, or insomnia (Olatunji et al., 2023). The relief of test and class anxiety has alleviated students' psychological pressure, enabling them to shift their focus and energy toward personal health and wellbeing. This shift has led many to actively engage in positive behaviors such as regular exercise and healthy eating. Psychological research suggests that health-related behaviors—including a balanced diet, controlled exercise, and adequate sleep—are linked to enhanced emotional wellbeing and a higher quality of life (Zeidner et al., 2012). Therefore, the improvement of social and emotional skills can promote students to have healthier lifestyles by alleviating test and class anxiety.

### 5.3 The moderating effect of satisfaction with the relationships on the interaction between social and emotional skills and health behavior

The results of this study indicate that the direct pathway through which social and emotional skills affect health behavior, as well as the first half of the mediating pathway via test and class anxiety, are moderated by satisfaction with relationships, thereby validating Hypothesis 3. Hypothesis 4 is partially supported. Specifically, compared to the group with low relationship satisfaction, the predictive effect of social and emotional skills on test and class anxiety was more pronounced in the group with high relationship satisfaction. In other words, higher satisfaction with relationships enhances the effectiveness of social and emotional skills in alleviating students' test and class anxiety. Good interpersonal relationships serve as a direct source of social support for adolescents. High satisfaction with these relationships typically indicates that students have a strong support network, providing them with a platform to share and express their emotions (Kim, 2015; Mzeno-Gutiérrez and López-Aguado, 2017). This, in turn, reduces their psychological burden and anxiety (Fu et al., 2022). In addition, good interpersonal relationships can help students develop more mature social and emotional skills (Yang et al., 2020), such as reasoning, trust, and respect for others. These skills are essential not only for building and maintaining relationships but also for helping students better understand and manage their emotions, ultimately alleviating test and class anxiety.

However, for the direct impact of social and emotional skills on health behavior, the moderation effect of low satisfaction with the relationships is more significant. This may be because adolescents with higher satisfaction with the relationships may invest more time in building and maintaining good interpersonal relationships. For example, teenagers use social media to establish and strengthen identification and connections with peer groups, and set boundaries for acceptance or exclusion (Charoensukmongkol, 2018). Although these activities are beneficial for maintaining interpersonal relationships, they may also distract their attention and practice of health behavior, thereby affecting their health behavior performance (Shao et al., 2022). In addition, the social environment of teenagers also has significant implications for their health behavior performance. Teenagers may engage in more negative behaviors, including drinking, through imitation, reinforcement, and other means in their social interactions with negative peers (Dodge et al., 2006). In this situation, even if adolescents have high interpersonal satisfaction with the relationships, they cannot exhibit health behaviors. Therefore, while social and emotional skills positively impact health behavior, the moderating role of relationship satisfaction underscores the importance of considering both the social environment and students' social needs when promoting health behavior. Efforts should focus on fostering a supportive social environment and improving social and emotional skills to ensure balanced development in both social and health aspects.

Regarding the non-significant moderating effect of relationship satisfaction on the path from test anxiety to health behavior (the second stage of the mediation), our findings suggest a potential boundary condition for the buffering role of social support. This non-significance can be theorized through the lens of cognitive load and resource depletion. High levels of test and class anxiety are not merely emotional states but also consume significant cognitive resources, leading to executive function impairment and ego depletion (Eysenck et al., 2007). This depleted state may directly undermine the self-regulatory capacity necessary for initiating and maintaining health behaviors, such as planning a healthy meal or resisting the impulse to skip exercise (Baumeister et al., 2007). When adolescents are cognitively exhausted by persistent academic anxiety, they may lack the mental strength to engage in effortful health-promoting activities, regardless of the perceived quality of their social relationships. Furthermore, the nature of the stressor is crucial. Satisfaction with interpersonal relationships may be more potent in mitigating the social-emotional origins of anxiety than in counteracting the direct cognitive and physiological consequences of anxiety which are more proximal determinants of health behavior execution (Bolger and Amarel, 2007). Therefore, our non-significant finding highlights that while supportive relationships are vital for preventing and reducing anxiety, their power to moderate the behavioral sequelae of established, high-intensity anxiety might be limited. This insight points to the necessity of interventions that directly target anxiety reduction and build self-regulatory skills, rather than solely relying on social support to buffer its downstream effects on health.

## 6 Contribution and implications

### 6.1 Contribution

The contribution of this study lies in its in-depth analysis of the complex relationship between adolescents' social and emotional skills and health behavior. Specifically, this study advances extant theories and practice in two ways. First, it integrates dual mechanisms—mediation and moderation—into a unified framework, addressing a gap in prior research that often treats these pathways in isolation. While existing studies have examined social and emotional skills as static predictors of outcomes, our findings reveal that their influence is context-dependent: the indirect effect of social and emotional skills on health behavior (via reduced test and class anxiety) is amplified among adolescents with higher relationship satisfaction. This underscores the dynamic interplay between individual skills and relational environments, a dimension underexplored in traditional social and emotional frameworks. Second, this study emphasizes the critical role of interpersonal relationships as moderators, demonstrating that relationship satisfaction not only enhances the efficacy of social and emotional in alleviating anxiety but also directly shapes how these skills translate into healthier behaviors. Prior research has largely focused on skill acquisition (e.g., self-regulation) while neglecting how relational contexts enable or constrain their application. By bridging this gap, our findings highlight the necessity of fostering supportive social environments alongside skill development—a dual focus essential for holistic interventions. Collectively, these contributions enrich the theoretical framework of social-emotional learning and provide actionable insights for designing programs that synergistically target both individual competencies and relational ecosystems to promote adolescent wellbeing.

### 6.2 Implication

#### 6.2.1 Developing social and emotional skills for adolescents

The cultivation of social and emotional skills has emerged as a global priority in the 21st century. According to this study, enhancing the social and emotional skills of adolescents can be a key measure to promoting their health behavior performance. Specifically, there are two main paths for cultivating social and emotional skills: one is to design specialized social and emotional learning courses or programs, and the other is to integrate the cultivation of social and emotional skills into daily curriculum teaching. SEL courses or programs serve as key tools for enhancing adolescents' social and emotional skills (Blanka, 2024). Through various activities, experiences, and structured learning, teenagers can systematically engage with core social-emotional competencies such as self-awareness, self-management, empathy, and collective cognition. This helps them develop both individual and interpersonal skills (Carroll et al., 2023; Xiao et al., 2025). In addition to specialized courses, incorporating the cultivation of social and emotional skills into the daily teaching system is also an important path (Gimbert et al., 2023). In teaching practice, teachers can adopt diverse teaching strategies such as cooperative learning, role-playing, and emotional education discussions, which can encourage students to learn to listen, respect, and understand others' perspectives through interaction.

#### 6.2.2 Relieving test and class anxiety among adolescents

In contemporary education, the testing and class anxiety of adolescents has become an issue that cannot be ignored. This anxiety not only affects their physical and mental health, but also has a profound impact on their academic performance and future career development (Hasty et al., 2023). The pervasive issue of test and class anxiety among adolescents necessitates a strategic approach that transcends conventional academic interventions, emphasizing the integration of social and emotional skills as a cornerstone in mitigating academic stress. Our study's implications align with a global shift toward curricular reformulations that prioritize emotional intelligence and socio-emotional skills. Educational practices must evolve to encompass SEL within curricula, providing adolescents with the tools to navigate anxiety effectively. Emotional education is pivotal, equipping students with strategies for stress management and emotional regulation, which are critical for mitigating the impact of academic pressures. The cultivation of psychological resilience through SEL programs not only buffers against academic anxiety but also fosters a growth mindset, where students perceive challenges as opportunities for growth rather than sources of distress. A supportive learning environment that promotes these skills can significantly reduce test-related anxiety, enhancing students' overall wellbeing and academic performance. Furthermore, the incorporation of mindfulness techniques, study skills enhancement, and peer support programs within school interventions can offer immediate and long-term benefits in managing anxiety. Engaging parents in their children's emotional development and promoting healthy lifestyle practices further amplify the effectiveness of these interventions. Collectively, these strategies underscore the imperative for schools to adopt a holistic approach to education that addresses the emotional and psychological needs of adolescents, thereby fostering a resilient and healthy student population.

#### 6.2.3 Building harmonious interpersonal relationships among adolescents

Building harmonious interpersonal relationships is crucial for the personal development and social adaptation of adolescents. Our study highlights the need for educational institutions to foster an inclusive culture that celebrates diversity and equips students with the skills to navigate complex social interactions. By modeling empathetic and respectful behaviors, educators can guide adolescents in developing critical competencies for positive relationship building. Extracurricular activities should be encouraged as they provide opportunities for students to form supportive peer groups and practice social skills in a nurturing environment. Additionally, engaging families in their children's social development through school-initiated programs can create a cohesive support system that extends beyond the classroom. Collectively, these efforts can nurture the interpersonal skills of adolescents, setting a foundation for successful social interactions and personal growth.

## 7 Limitations and future research direction

Although this study reveals the impact and mechanism of adolescents' social and emotional skills on health behavior, there are still some limitations. First, the cross-sectional nature of the data prevents definitive causal inferences. Although our model is theoretically grounded, the relationships between social and emotional skills, anxiety, and health behavior are likely bidirectional. Future research should employ longitudinal or experimental designs to establish causality and explore the dynamic, reciprocal nature of these variables over time. Secondly, the study does not include a comparative analysis across different cultural, demographic, or socioeconomic groups. This limitation restricts the generalizability of our findings, as the sample is drawn from a specific city in China and may not fully represent the diverse experiences of adolescents in other regions or socioeconomic contexts. Future research should incorporate comparative analyses to explore how cultural, demographic, and socioeconomic factors influence the relationship between social and emotional skills and health behavior. Thirdly, regarding the analytical method, this study utilized a regression-based approach (PROCESS macro) to test the moderated mediation model with observed composite scores. While this method is robust and well-established for testing such hypotheses, Structural Equation Modeling (SEM) would offer distinct advantages. Specifically, a full SEM framework with latent variables could simultaneously account for measurement error in the constructs and provide a more comprehensive test of the entire model fit. Future research would benefit from employing SEM to replicate and extend our findings, thereby providing an even more rigorous examination of the complex relationships between social-emotional skills, anxiety, interpersonal relationships, and health behaviors. In addition, although this study explored the influence of social and emotional skills and moderating effect of satisfaction with the interpersonal relationships, it did not delve into the impact of different types of sub-skills (e.g., emotional regulation, task performance) and interpersonal relationships (e.g., family, peer, and teacher-student relationships) on adolescent health behavior. Future research could employ a more granular approach to identify which dimensions of social and emotional skills are most predictive of health outcomes, and under what relational or contextual conditions.

## Data Availability

Publicly available datasets were analyzed in this study. This data can be found here: https://www.oecd.org/en/data/datasets/SSES-Round-2-Database.html.
